# Serum Amyloid A Truncations in Type 2 Diabetes Mellitus

**DOI:** 10.1371/journal.pone.0115320

**Published:** 2015-01-21

**Authors:** Hussein N. Yassine, Olgica Trenchevska, Huijuan He, Chad R. Borges, Dobrin Nedelkov, Wendy Mack, Naoko Kono, Juraj Koska, Peter D. Reaven, Randall W. Nelson

**Affiliations:** 1 University of Southern California, Los Angeles, CA, United States of America; 2 Arizona State University, Tempe, AZ, United States of America; 3 Phoenix VA Health Care System, Phoenix, AZ, United States of America; University of Michigan Medical School, UNITED STATES

## Abstract

Serum Amyloid A (SAA) is an acute phase protein complex consisting of several abundant isoforms. The *N*- terminus of SAA is critical to its function in amyloid formation. SAA is frequently truncated, either missing an arginine or an arginine-serine dipeptide, resulting in isoforms that may influence the capacity to form amyloid. However, the relative abundance of truncated SAA in diabetes and chronic kidney disease is not known.

**Methods:**

Using mass spectrometric immunoassay, the abundance of SAA truncations relative to the native variants was examined in plasma of 91 participants with type 2 diabetes and chronic kidney disease and 69 participants without diabetes.

**Results:**

The ratio of SAA 1.1 (missing *N*-terminal arginine) to native SAA 1.1 was lower in diabetics compared to non-diabetics (p = 0.004), and in males compared to females (p<0.001). This ratio was negatively correlated with glycated hemoglobin (r = −0.32, p<0.001) and triglyceride concentrations (r = −0.37, p<0.001), and positively correlated with HDL cholesterol concentrations (r = 0.32, p<0.001).

**Conclusion:**

The relative abundance of the *N*-terminal arginine truncation of SAA1.1 is significantly decreased in diabetes and negatively correlates with measures of glycemic and lipid control.

## Introduction

Serum Amyloid A (SAA) proteins are members of the acute phase response protein group that play a significant role in inflammatory processes [1]. Structurally, proteins in the SAA complex are encoded by four genes; SAA 1 and SAA 2 genes code acute phase SAA protein isoforms, SAA 3 is not expressed in humans, while SAA 4 is constitutively expressed under homeostatic conditions but does not vary significantly in the acute phase [2]. SAA 1 and SAA 2 genes give rise to five SAA 1 (SAA 1.1, SAA 1.2, SAA 1.3, SAA 1.4 and SAA 1.5) and two SAA 2 (SAA 2.1 and SAA 2.2) protein isoforms, respectively. All these protein products share a high degree of homology and differ only by a few amino acids in their sequences. Only SAA 1.1, SAA 1.2, SAA 1.3, SAA 2.1 and SAA 2.2 have been identified in humans [3] .

Following an inflammatory stimulus, SAA proteins act as apolipoproteins and become a major component of high density lipoproteins (HDL). This process removes cholesterol and extracellular lipid deposits at the inflammation site, but can reduce the ant-inflammatory capacity of HDL [4–6]. SAA is also chronically elevated in the setting of secondary amyloidosis leading systemic deposition of SAA-derived truncated fibrils (AA proteins) in tissues [7]. Secondary amyloidosis has been associated with chronic inflammatory conditions such as rheumatoid arthritis [8] or multiple sclerosis [9].In addition, SAA is recognized as a potential biomarker for a variety of chronic diseases including diabetes, cardiovascular disease (CVD), different types of cancers, sickle-cell disease, traumatic tissue damage and infections [10–13].

The role of SAA in each of these many conditions is generally believed to be a consequence of increases in total plasma concentration. However, not all studies find associations between disease and concentrations of total SAA. The discrepant results may be accounted for by the fact that most SAA studies do not discriminate between different molecular species of SAA, and do not consider the posttranslational modifications of the protein [14]. In fact, some studies demonstrate variations in the relative abundance of SAA truncated at the *N*-terminus [3,15,16]. This may have important clinical implications as the *N*-terminus of SAA is critical for amyloid fibrils formation [17,18]. The clearance of this truncated SAA variant is different compared to native SAA suggesting that these truncations may affect the capacity to form amyloid [16]. Therefore, it would be important to study if the relative abundances of the truncated forms differ in patients with chronic conditions such as diabetes or chronic kidney disease (CKD).

Current analytical techniques for SAA based on conventional immunoassay approaches provide total protein quantification. However, several mass-spectrometry based methods can distinguish between the different protein variants and posttranslational SAA protein modifications [12,19,20] . Mass spectrometric immunoassay (MSIA) is a high throughput methodology that is well suited to identify and quantify molecular variants and posttranslational modifications of plasma proteins [21,22]. MSIA is based on the isolation of protein moieties from a biological milieu by immobilized antibodies, which is followed by mass spectrometry assay. In our previous work using MSIA, we identified several novel *N*-terminal truncations of SAA 1.1, SAA1.2 and SAA 2.1 in human plasma [3]. In the present study, we investigate the association of SAA truncations with type 2 diabetes and chronic kidney disease (CKD).

## Materials and Methods

### Clinical Samples

The study was approved by the University of Arizona Institutional Review Board, and all patients provided written informed consent prior to testing. Two groups of adult participants (>18 years of age) were recruited: participants with type 2 diabetes (n = 91; 47 males and 44 females) and non-diabetic controls (n = 69, 29 males and 40 females). Participants reported to the Center for Clinical and Translational Sciences after an overnight fast. Blood was collected for clinical laboratory measurements (lipid profile, HbA1c, CRP, fasting insulin). Additional samples were collected in EDTA tubes, and plasma from these samples was separated and immediately frozen at −80°C for all other assays. Demographic information (age, sex, ethnicity), physical exam measurements (blood pressure, waist circumference, weight, height, body mass index), medication use, and medical history (hypertension, hyperlipidemia, smoking, type and duration of diabetes) were also recorded. The glomerular filtration rate (GFR) was estimated using the Modification of Diet in Renal Disease Study (MDRD) equation [23]. Assignment of CKD (stages 1–5) was based on GFR levels as described [24]. Exclusion criteria included the following: type 1 diabetes, participation in an active weight loss program, history of cancer, HIV, active infection, other ongoing serious illness or current steroid use. Diabetes classification was based on clinical and medication history, or glycated hemoglobin greater than 6%. For the SAA MSIA analysis, frozen plasma samples were briefly thawed on ice, centrifuged for 5 min at 3000 rpm, and 50 μL aliquoted in low profile 96-well trays. Prior to the analysis, samples were diluted with 100 μL PBS containing 0.1%Tween.

### Reagents

Monoclonal mouse anti-human antibody to serum amyloid A (SAA) was obtained from ANOGEN (Mississauga, ON, Canada, Catalog No. MO-C40028A, 500 mg). Protein calibration standard I (Cat. No. 206355) was purchased from Bruker (Billerica, MA). Phosphate buffered saline (PBS) buffer (Cat. No. 28372), MES buffered saline (28390), 1, 1’carbonyldiimidazole (97%) (CDI, 530-62-1), acetonitrile (ACN, A955-4), hydrochloric acid (HCl, A144-212), *N*-Methylpyrrolidinone (NMP, BP1172-4) and affinity pipettes fitted with porous microcolumns (991CUS01) were obtained from Thermo Scientific (Waltham, MA, USA). Tween 20 (Cat. No. P7949), trifluoroacetic acid (TFA, 299537-100G), sinapic acid (85429-5G), sodium chloride (S7653), HEPES (H3375), ethanolamine (ETA, 398136), albumin from bovine serum (BSA, A7906), ammonium acetate (A7330), octyl β-D glucopyranoside (NOG, 08001), polyethylene glycol (P3015-250G), calcium chloride (C1016-100G), and glycine buffer (G8898-500G) were obtained from Sigma Aldrich (St. Louis, MO, USA). Acetone (Cat. No. 0000017150) was obtained from Avantor Performance Materials (Center Valley, PA, USA). Plasma SAA was measured by an ELISA Kit (Novex, Cat. No. KHA0012).

### Mass spectrometric immunoassay affinity pipette derivatization

The first step in MSIA method development is derivatization of the affinity pipettes with corresponding antibody toward the targeted protein. Using a Multimek 96 pipettor (Beckman Coulter, Brea, CA ) affinity pipettes were derivatized with antibody toward SAA by initial rinse with 200 mM HCl (2 × 20 cycles, each cycle consisting of an aspiration and dispense of a 120 μL volume), followed by an acetone rinse (1×20 cycles). The surface of the microcolumns was activated by immersing the pipettes into a tray containing 100 mg/mL 1, 1’carbonyldiimidazole (in *N*-Methylpyrrolidinone), and 450 cycles of 100 μL aspirations and dispenses through each of the affinity pipettes were performed. Two rinses with NMP (10 cycles each, 150 μL volume) followed. The affinity pipettes were then immersed into a microwell plate containing the antibody solution (5 μg Ab per tip) and 750 cycles of aspirations and dispenses of 50 μL volume were performed to bind the antibody to the activated surfaces. One rinse with 60 mM HCl followed (50 cycles each, 100 μL), ending with two final rinses with assay buffer (PBS w/0.1% Tween, 50 cycles each, 100 μL). Antibody-derivatized pipettes were stored at +4°C until used.

### Mass spectrometric immunoassay

Prior to sample protein extraction, the derivatized affinity pipettes were pre-rinsed with assay buffer (PBS, 0.1%Tween, 50 aspiration/dispense cycles, 100 μL each), followed by two 60 mM HCL rinses (50 aspiration/dispense cycles, 100 μL each) and another assay buffer rinse. Based on prior experiments, pre-rinsing reduces non-specific binding to the tips during protein extraction. Pipettes were then immersed into a microplate containing the samples and 250 aspirations and dispense cycles were performed (100 μL each) allowing for affinity capture of SAA from the samples. The pipettes were then rinsed with mixture of 1 M ammonium acetate and acetonitrile (AA: ACN = 3:1 (v/v)) (100 cycles), and twice with water (10 cycles and 20 cycles respectively, 100 μL aspiration/dispense each). SAA-loaded tips were then exposed to six-microliter aliquots of MALDI matrix solution (25 g/L sinapic acid in aqueous solution containing 33% (v/v) acetonitrile, and 0.4% (v/v) trifluoroacetic acid). After a 10 second delay (to allow for the dissociation of the protein from the capturing antibody), the eluates were dispensed directly onto a 96-well formatted MALDI target. Following drying, linear-mode mass spectra were acquired from each sample spot, each consisting of ten thousand laser shots using an *Autoflex* III MALDI-TOF mass spectrometer (Bruker, Billerica, MA). The mass spectra were internally calibrated using low molecular mass protein standards, and further processed (baseline subtracted and smoothed) with the Flex Analysis software (Bruker Daltonics). Antibody specificity was verified by confirming the absence of SAA-related mass spectral peaks from negative control tips on which 1) no antibody was present, and 2) on which monoclonal antibodies specific to other proteins were immobilized. The peak areas for all SAA signals were measured in Zebra software (Beavis Informatics, Ltd.). Relative percent abundance of every SAA isoform was calculated in correspondence to the total SAA in the corresponding mass spectra.

### SAA quantification

Total SAA concentrations were measured using a commercial ELISA from Invitrogen Life technologies (Novex, Cat. KHA0012) that has been extensively used [25–29] and tested on serum, plasma and tissue culture samples. The kit is based on antibody affinity to SAA 1.1, and thus does not capture the other forms of SAA.

## Statistical Analysis

Mean (SD) or median (25th and 75th percentiles) were calculated for continuous variables. The diabetes and non-diabetes groups were compared by independent t-tests (normally distributed variables) or Wilcoxon rank sum tests (non-normally distributed variables). Categorical variables were compared using the chi-square test. The relationship between SAA variants and diabetes status was measured by logistic regression, with diabetes status as the binary dependent variable. The relation between SAA truncations and clinical variables was modeled using linear regression; all regression models used log-transformed SAA 1.1R as the dependent variable, and included age, BMI, and GFR as adjusting covariates. Regression models were performed in the total sample and by gender; a product interaction term tested whether the associations of clinical variables with SAA variants differed in males vs. females. Statistical analyses used SAS version 9.3 software package; a p-value of < 0.007 (0.05 divided by 7) was used to define significance levels in the SAA variants between the diabetes and non-diabetes groups accounting for the seven SAA variants (SAA 1.1R, SAA 1.1RS, SAA 2.1R, SAA 2.1 RS, SAA 2.2R, SAA 1.3R and SAA 1.3 RS truncations) of interest in this study. All other statistical tests used a p-value of 0.05.

## Results

Participants’ demographic and biochemical characteristics are summarized in Table 1. Participants with diabetes were on average older, had greater BMI, and demonstrated several metabolic characteristics of type 2 diabetes, including low HDL cholesterol, increased fasting insulin and elevated CRP levels when compared to participants without diabetes. In addition, participants with diabetes had a lower GFR and a greater urine albumin excretion (p<0.01 for both) compared with the non-diabetic controls. Males (in the combined group) had significantly lower GFR than females (mean ± SD: male: 85.6 ± 32.5 L/min/1.73m^2^; female: 98.4 ± 35.6 L/min/1.73m^2^, p = 0.03).

**Table 1:** Demographic and clinical characteristics.

Characteristic	N_1_/ N_2_	No diabetes	Diabetes	p-value
Age, years	**69 / 91**	**49.4 (15.1)**	**56.5 (12.8)**	**0.002**
Sex	69 / 91			
Male		29 (42.0%)	47 (51.6%)	0.23
Female		40 (58.0%)	44 (48.4%)	
Race	69 / 91			
Caucasians		45 (65.2%)	50 (54.9%)	0.41
Hispanics		21 (30.4%)	35 (38.5%)	
Others		3 (4.4%)	6 (6.6%)	
Body mass index, kg/m^2^	69 / 86	**27.8 (24.4, 32.8)**	**33.9 (28.2, 39.6)**	**<0.001**
Waist circumference, cm	68 / 80	**98.2 (17.2)**	**115.0 (19.8)**	**<0.001**
Glucose, mg/dL	69 / 85	**101.0 (93.0, 110.0)**	**144.0 (118.0, 202.0)**	**<0.001**
Hemoglobin A1C, (%)	65 / 77	**5.3 (5.1, 5.8)**	**7.8 (6.5, 9.8)**	**<0.001**
LDL cholesterol, mg/dL	68 / 84	117.3 (33.1)	107.7 (38.4)	0.11
HDL cholesterol, mg/dL	68 / 84	**52.6 (14.2)**	**45.2 (12.3)**	**<0.001**
Total cholesterol, mg/dL	68 / 84	196.6 (38.7)	186.1 (45.7)	0.14
Triglycerides, mg/dL	68 / 84	**131.5 (97.5, 182.5)**	**163.5 (134.0, 267.0)**	**0.001**
Glomerular FiltrationRate (GFR), L/min/1.73 m^2^	65 / 76	**104.9 (25.1)**	**81.6 (38.1)**	**<0.001**
Fasting Insulin, IU		**9.0 (7.0, 14.0)**	**16.0 (8.0, 26.0)**	**0.004**
Urine Microalbumin,mg/mg creatinine	66 / 78	**5.0 (5.0, 9.0)**	**12.5 (5.0, 61.0)**	**<0.001**
Diabetes duration, years	0 / 85	-	9.0 (4.0, 15.0)	-
CRP, mg/dL	34 / 64	**2.8 (1.0, 6.1)**	**6.8 (3.0, 12.1)**	**0.006**
Total SAA, ng/mL*	21 / 21	28.7 (17.3, 44.5)	21.2 (9.9, 38.6)	0.21

Mean (SD) or median (25^th^ percentile, 75^th^ percentile) shown for continuous variables (normally distributed or non-normal, respectively).

*Only a subset from the whole sample was evaluated for total SAA concentration by ELISA.

Total SAA concentration was evaluated using a commercial ELISA in an unselected subset of samples (non-diabetes n = 21 vs. diabetes n = 21). There was no significant difference in total SAA concentration between the non-diabetes and diabetes groups: (median (25^th^ percentile, 75^th^ percentile), non-diabetes: 28.7 (17.3, 44.5), diabetes: 21.2 (9.9, 38.6), p = 0.21).

**Figure 1:**
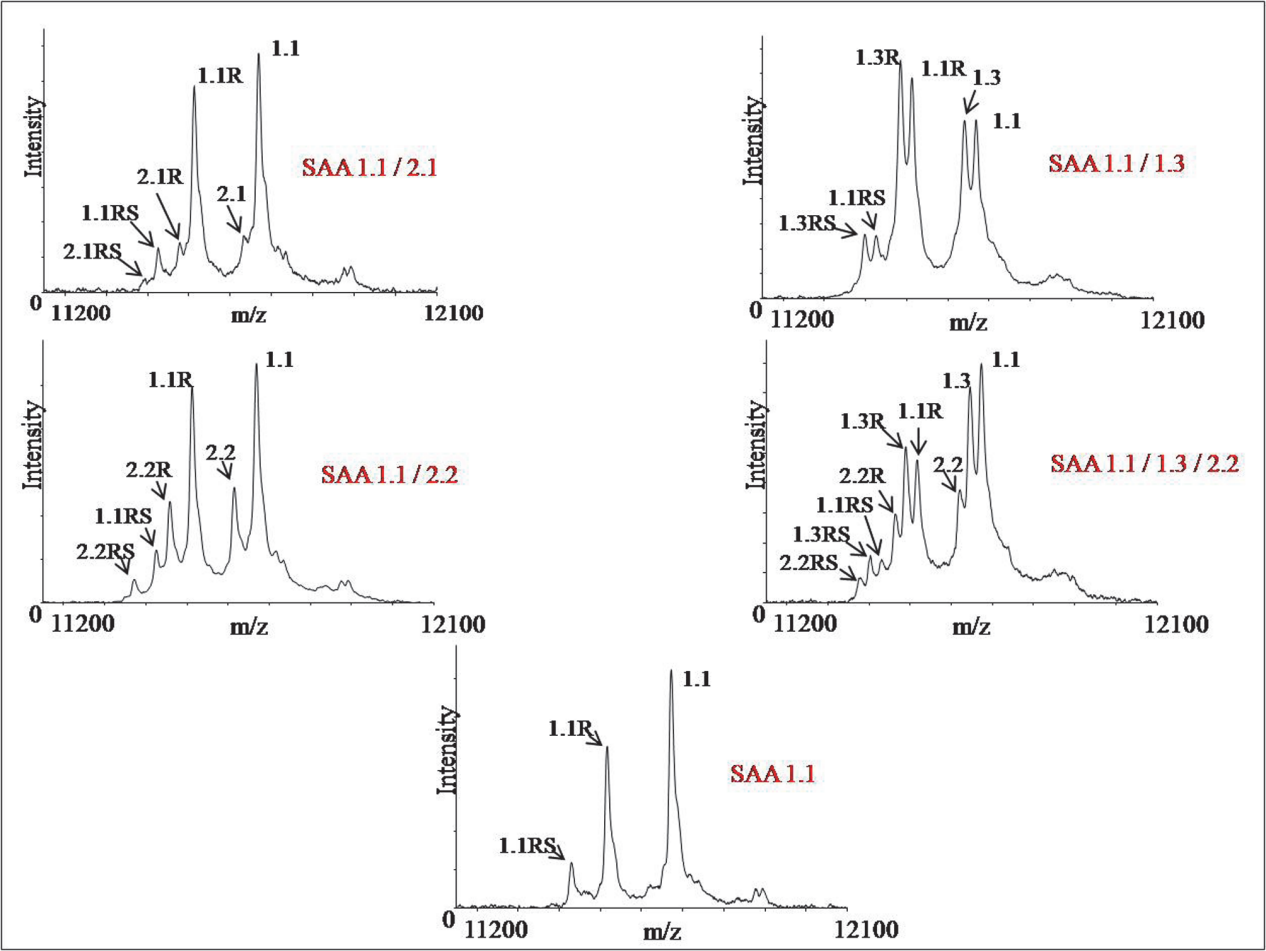
MALDI-MSIA-mass spectra of SAA from five different plasma samples showing all the variant forms and truncations (missing terminal –R and/or terminal –RS). All samples express SAA 1.1 with R and RS truncations.

Characteristics of known SAA variants are summarized in Table 2. The SAA variants that can be resolved by MSIA are summarized in Table 3. A total of four protein isoforms, SAA 1.1, SAA 1.3, SAA 2.1 and SAA 2.2 (reflecting protein products from SAA1 and SAA2 genes) were detected among the samples, expressed either as single or as multiple variants. MALDI-MSIA-mass spectra of SAA from five different plasma samples showing all the variant forms and truncations (missing terminal –R and/or terminal –RS) are shown in Fig. 1. All samples that expressed SAA 1.1, presented with R and RS truncations. The MSIA analysis confirmed that the SAA variant 1.1 (MW = 11682.68) was the most abundant and it was detected in all but one (159/160) individual. The distribution of the other identified SAA protein entities within the cohort was as follows: 58.8% for SAA 2.1, 13.1% for SAA 2.2 and 11.9% for SAA 1.3. In addition to these native forms, two *N*-terminal truncation isoforms were noted for all the variants: those missing arginine (des-R; Δm = −156.19 Da) and/or arginine-serine dipeptide (des-RS; Δm = −243.27 Da).

**Table 2:** SAA isoforms detected by MSIA.

**SAA gene**	**SAA proteins**	**Amino acid sequence**	**native form**	-**R** **truncated form**	-**RS** **truncated form**
Allelic genes			MW	MW	MW
*SAA1*	SAA 1.1	RS FFSFLGEAFD GARDMWRAYS DMREANYIGS DKYFHARGNY DAAKRGPGGV WAAEAISDAR ENIQRFFGHG AEDSLADQAA NEWGRSGKDP NHFRPAGLPE KY	11682.7	11526.5	11439.4
	SAA 1.2	RS FFSFLGEAFD GARDMWRAYS DMREANYIGS DKYFHARGNY DAAKRGPGG**A** WAAEVISDAR ENIQRFFGH**D** AEDSLADQAA NEWGRSGKDP NHFRPAGLPE KY	11740.7	11584.5	11497.5
	SAA 1.3	RS FFSFLGEAFD GARDMWRAYS DMREANYIGS DKYFHARGNY DAAKRGPGG**A** WAAEAISDAR ENIQRFFGHG AEDSLADQAA NEWGRSGKDP NHFRPAGLPE KY	11654.6	11498.4	11411.4
	SAA 1.4*	RS FFSFLGEAFD GARDMWRAYS DMREANYIGS DKYFHARGNY DAAKRGPGG**A** WAAEVISNAR ENIQRFFGHG AEDSLADQAA NEWGRSGKDP NHFRPAGLPE KY	11681.7	11525.5	11438.4
	SAA 1.5*	RS FFSFLGEAFD GARDMWRAYS DMREANYIGS DKYFHARGNY DAAKRGPGG**A** WAAEVISDAR ENIQRFFGHG AEDSLADQAA NEWGRSGKDP NHFRPAGLPE KY	11682.7	11526.5	11439.4
*SAA2*	SAA 2.1	RS FFSFLGEAFD GARDMWRAYS DMREANYIGS DKYFHARGNY DAAKRGPGGA WAAEVISNAR ENIQRLTG**H**G AEDSLADQAA NKWGRSGRDP NHFRPAGLPE KY	11628.7	11472.5	11385.4
	SAA 2.2	RS FFSFLGEAFD GARDMWRAYS DMREANYIGS DKYFHARGNY DAAKRGPGGA WAAEVISNAR ENIQRLTGRG AEDSLADQAA NKWGRSGRDP NHFRPAGLPE KY	11647.7	11491.5	11404.5
*SAA4*	SAA 4**	ES WRSFFKEALQ GVGDMGRAYW DIMISNHQNS NRYLYARGNY DAAQRGPGGV WAAKLISRSR VYLQGLIDCY LFGNSSTVLE DSKSNEKAEE WGRSGKDPDR FRPDGLPKKY	12802.2		

SAA exists in multiple forms. The main isoforms expressed are SAA 1.1 and 2.2. MSIA can detect the SAA 1.1, 1.2, 1.3, 2.1 and 2.2. SAA 1.4 and 1.5 cannot be resolved. SAA 3 is a pseudo gene (not expressed). SAA 4 is constitutively expressed. MSIA: Mass Spectrometric Immunoassay.

* These variants can’t be distinguished in the mass spectra due to close proximity (overlapping) of the mass in spite of the changes in the amino acid sequence.

** SAA 4 is constitutively expressed.

**Table 3:** Comparison of SAA variant ratios between the diabetes and non-diabetes groups.

**SAA Variant Ratio**	**% variants detected**	**N_1_ / N_2_**	**Non-Diabetes**	**Diabetes**	**p-value**
SAA 1.1R	99.4% (159/160)	69 / 90	0.93 (0.76, 1.17)	0.84 (0.65, 0.99)	0.004*
SAA 1.1RS	99.4% (159/160)	69 / 90	0.22 (0.18, 0.28)	0.21 (0.15, 0.29)	0.11
SAA 2.1R	57.5% (92/160)	34 / 58	0.88 (0.76, 1.10)	0.90 (0.78, 1.04)	0.73
SAA 2.1RS	29.4% (47/160)	16 / 31	0.25 (0.20, 0.30)	0.25 (0.20, 0.32)	0.96
SAA 2.2R	13.1% (21/160)	6 / 15	0.70 (0.53, 0.89)	0.81 (0.76, 0.93)	0.32
SAA 1.3R	11.9% (19/160)	6 / 13	0.79 (0.74, 1.24)	0.96 (0.80, 1.18)	0.94
SAA 1.3RS	10.6% (17/160)	5 / 12	0.23 (0.23, 0.27)	0.28 (0.22, 0.36)	0.42

SAA isoforms were heavily truncated at the *N* terminus missing either arginine (R) or arginine serine (RS). The truncation abundance and the differences in participants with diabetes and without diabetes are summarized. The data are presented as medians (25^th^ percentile, 75^th^ percentile) by diabetes status. The comparison between the two groups was performed with a logistic regression model after adjusting for age.

*Indicates statistically significant at the threshold of 0.007. After adjusting for age and BMI, the association of diabetes and SAA 1.1R was significant at the .05 alpha level: OR(95 CI) = 0.24 (0.07 to 0.78), p = 0.02.

**Figure 2:**
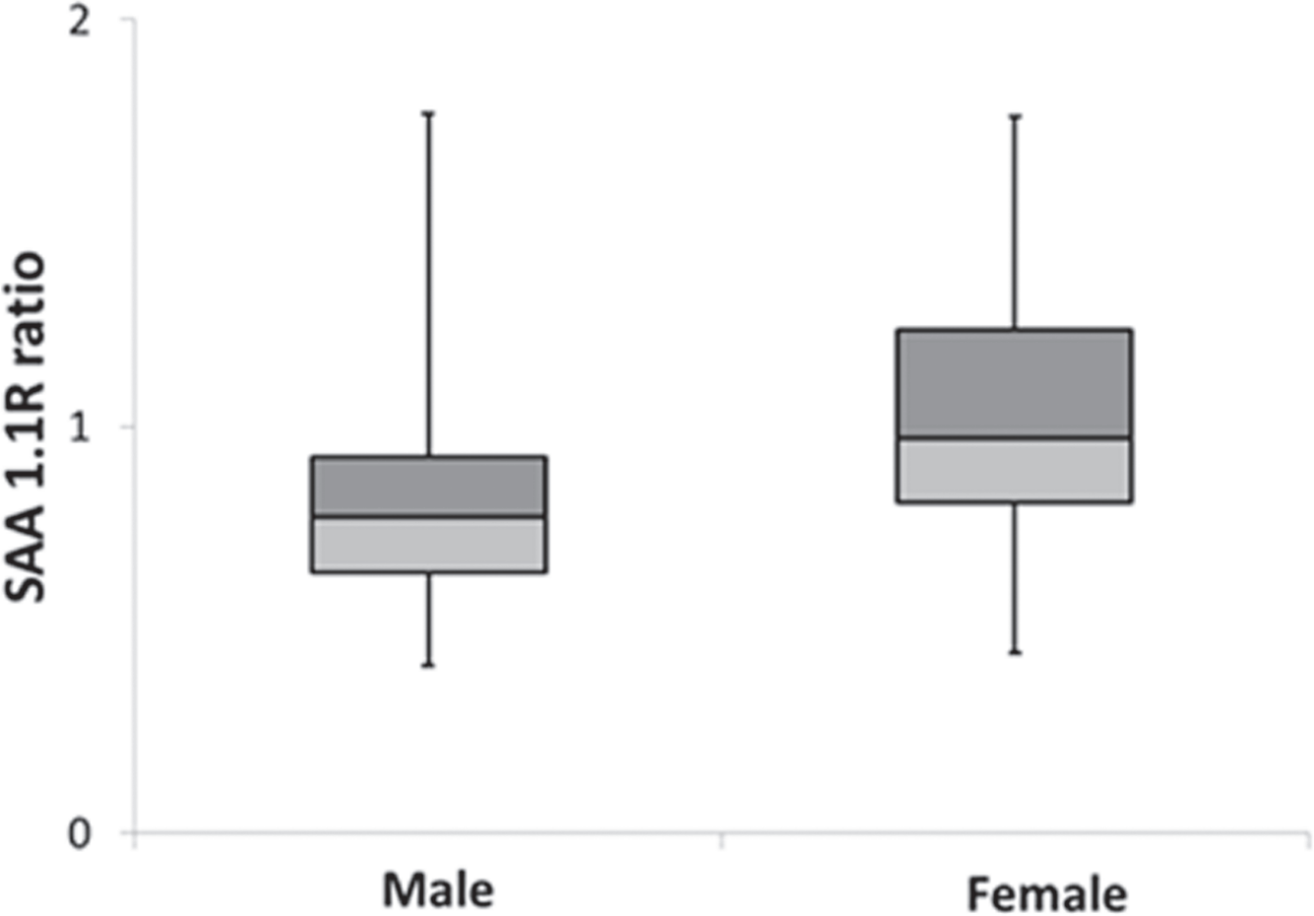
SAA variant ratios (median, IQR) among males (n = 76) and females (n = 84) group. SAA 1.1R (missing *N*-terminal R arginine) ratio to SAA 1.1 was significantly lower in males compared to females (p<0.001).

The relative abundance of each of the truncated isoforms was calculated as the ratio of truncated SAA to native SAA and compared between subjects with and without diabetes (SAA variants R and RS ratios). The log of SAA1.1R ratio was higher in the non-diabetic group compared to the diabetic group, (p = 0.02). The log of SAA1.1R was significantly associated with age (r = 0.16, p = 0.03), but not with BMI (r = −0.12, p = 0.11). As shown in Table 3, after adjustment for age, SAA 1.1R was lower in the diabetes group compared with the non-diabetes group (p = 0.004); the association remained significant with adjustment for both BMI and age (p = 0.02). In contrast, the ratio of other SAA truncation variants to native (SAA 1.1RS, 2.1R, 2.1RS, 2.2R, 1.3R and 1.3 RS truncations) did not differ between the diabetes and non-diabetes groups (all p>0.05, Table 3). In addition, in the whole group (diabetes and non-diabetes combined) the SAA 1.1R ratio to native was lower in males compared with females (median (IQR): male: n = 76, 0.78 (0.29); female: n = 84, 0.97 (0.43), p<0.001), Fig. 2).

**Figure 3:**
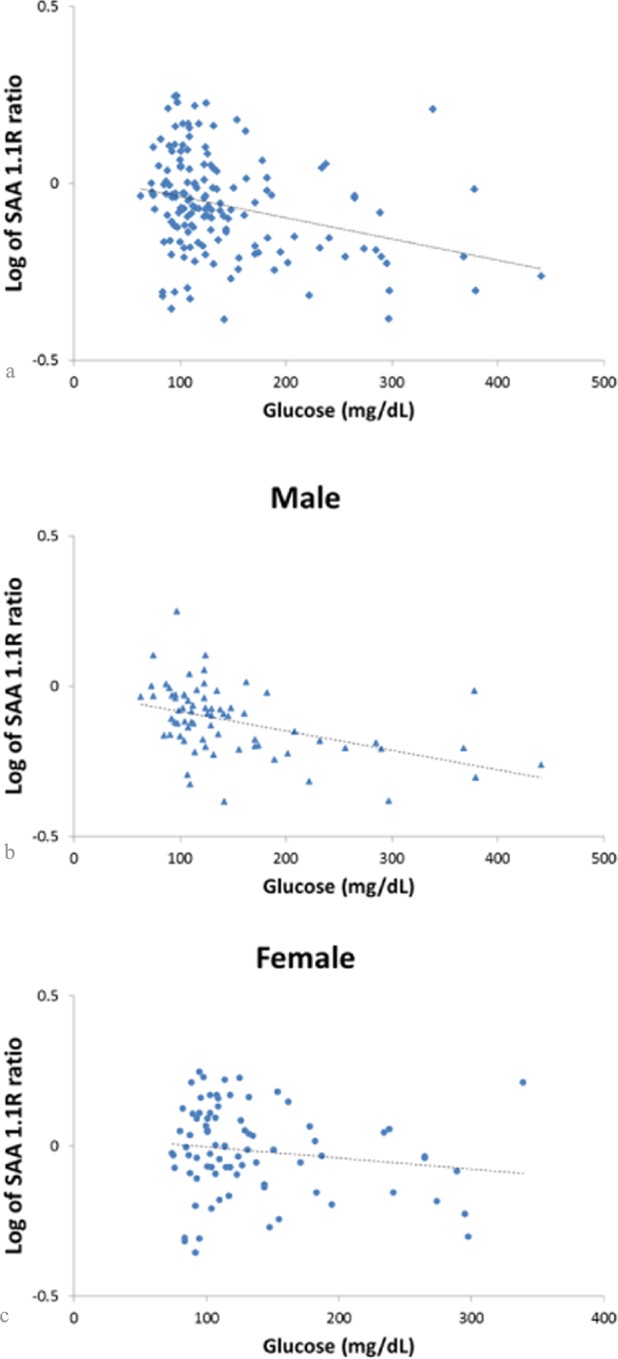
Correlation between glucose and the log of SAA 1.1R ratio in the entire group (panel a), in males (panel b), and in females (panel c). In the entire group, the log of SAA 1.1R was inversely related to glucose levels (panel a, r = −0.3, p<0.001). This correlation was driven by the males in the study. The log of SAA 1.1R was inversely correlated to glucose in males (panel b, r= −0.44, p<0.001), but not in females (panel c, r = −0.15, p = 0.17).

An inverse correlation was observed between glucose levels and log-transformed SAA 1.1R (r = −0.3, p<0.001). The correlation between log-transformed SAA1.1R and glucose was significant in males (r = −0.44, p<0.001, Fig. 3B) but not in females (r = −0.15, p = 0.17, Fig. 3C). Table 4 summarizes the linear associations of log-transformed SAA 1.1R ratio and several metabolic measures in males and females, adjusting for age, BMI and GFR. Both fasting glucose and glycated hemoglobin were strongly inversely correlated with log-transformed SAA 1.1R ratio. Although none of these associations significantly differed in males and females (all interaction p-values > 0.05), the SAA 1.1R ratio was most strongly and inversely correlated with glucose levels in males. There was no correlation between total SAA concentrations measured by ELISA and the SAA1.1R ratio determined by MSIA.

**Table 4:** Relationship of SAA 1.1R variant ratios with clinical and laboratory characteristics.

		**Combined sample**		**Male**		**Female**	
	**N/N_1_/N_2_**	**Regression Coefficient** **Beta (SE)**	**p-value**	**Regression Coefficient Beta (SE)**	**p-value**	**Regression Coefficient Beta (SE)**	**p-value**
Waist circumference, cm	129/59/70	**−0.008(0.003)**	**0.02**	−0.004(0.006)	0.47	−0.002(0.004)	0.59
Glucose,mg/dL	134/62/72	**−0.001(0.0004)**	**0.004**	**−0.002(0.0004)**	**<0.001**	−0.0006(0.0007)	0.41
Glycated Hemoglobin %	128/61/67	**−0.040(0.011)**	**<0.001**	**−0.035(0.010)**	**0.001**	−0.028(0.019)	0.15
Triglycerides,mg/dL	135/62/73	**−0.0006(0.0002)**	**0.01**	−0.0003(0.0002)	0.23	−0.0004(0.0004)	0.33
HDL,cholesterolmg/dL	135/62/73	**0.007(0.002)**	**0.003**	0.003(0.004)	0.43	0.002(0.003)	0.59
CRP,mg/dL	82/42/40	−0.001(0.006)	0.85	0.0005(0.006)	0.94	−0.005(0.008)	0.52

Linear regression with log-transformed SAA 1.1R as the dependent variable with clinical and laboratory characteristics as the independent variables adjusted for age, BMI and glomerular filtration rate (GFR).

All samples in table include both diabetics and non-diabetics.

N = number of subjects in combined sample (male and female subjects)

N_1_ = number of male subjects

N_2_ = number of female subjects

Since males had a lower GFR than females, the relative abundance of SAA truncations could be explained by differences in kidney clearance rate. To address this possibility, the relationship between log-transformed SAA 1.1R and GFR in both males and females was examined. In males, log-transformed SAA 1.1R was not significantly correlated with GFR (r = 0.21, p = 0.09); the association was reduced with adjustment for glucose (r = 0.12, p = 0.37). In females, log-transformed SAA1.1R and GFR were inversely but not significantly correlated (r = −0.221, p = 0.06); adjustment for glucose did not alter this association (r = −0.21, p = 0.08). These glucose-adjusted associations did not significantly differ among males and females (p-value for interaction = 0.09).

The study group was categorized by CKD stage with the non-CKD group defined based on a GFR > 90 L/min/1.73m^2^. The distribution of CKD stages in the diabetes and non-diabetes groups is presented in Table 5. There were 61 patients in the CKD group and 80 patients in the non-CKD group. The majority of non-diabetic participants did not have evidence of CKD, whereas the diabetic participants recruited in this study had different stages of CKD with 9 participants in stages 4 and 5. The inverse association of log-transformed SAA1.1R with glucose did not differ among persons with CKD vs. non-CKD (interaction p-value = 0.12, adjusted for age and BMI). These findings confirm that glucose levels had an inverse correlation with SAA 1.1R, with the association apparent in persons with and without CKD.

**Table 5:** Numbers of participants in non-diabetes and diabetes groups based on their CKD stages.

**CKD stage**	**Non-Diabetes N = 65**	**Diabetes N = 76**
Stage1 (GFR ≥90 mL/min/1.73m^2^)	46	34
Stage2 (60≤GFR<90 mL/min/1.73m^2^)	18	21
Stage3 (30≤GFR<60 mL/min/1.73m^2^)	1	12
Stage4 (15≤GFR<30 mL/min/1.73m^2^)	0	6
Stage5 (GFR<15 mL/min/1.73m^2^)	0	3

GFR values for 19 patients were unavailable.

Fisher’s exact test was used to test for differences in CKD stages between diabetics and non-diabetics, p < 0.001.

The peak area of SAA 1.1 was highly correlated to the peak area of SAA 1.1R and SAA 1.1RS (Table 6). The correlation was strongest (r> 0.9, p <0.001, all subjects) among the SAA 1.1 R and RS variants suggesting that the same process favors the formation of both truncations.

**Table 6:** Correlations between SAA 1.1 variants in diabetes group.

**Variants**	**All subjects** **N = 159**	**No Diabetes** **N_1_ = 69**	**Diabetes** **N_2_ = 90**
SAA 1.1 with SAA 1.1R	0.87	0.84	0.90
SAA 1.1 with SAA 1.1RS	0.81	0.78	0.83
SAA 1.1R with SAA 1.1RS	0.91	0.89	0.93

The peak area of log-transformed SAA 1.1 (unmodified) was highly correlated to the peak area of log-transformed SAA 1.1R and SAA 1.1RS (all p-values <0.001).

## Discussion

Our findings demonstrate that ratios of SAA 1.1R truncations to their native SAA variants are decreased in diabetes. The occurrence of *N*-terminally truncated SAA has been observed in several studies confirming that the relative abundance of this variant of SAA is quite high [3,12,15,16,30,31]. The population frequency of these truncations and their physiologic relevance are not known. Since the *N* terminus of SAA determines its amyloid fibril formation activity [17,18], assessing changes in SAA truncations might provide insight into diseases such as reactive amyloidosis where susceptibility to amyloid formation is dependent on the SAA functionality and clearance. We previously reported a lower relative abundance of SAA truncations in plasma of one subject with acute inflammation (rheumatoid arthritis) compared with subjects without evidence of inflammation [3]. Given that the *N*-terminal arginine and serine of SAA are highly conserved, these truncations are likely the result of posttranslational modifications [12,32]. Dipeptidyl aminopeptidase cleavage has been reported for several lipoproteins [33], and SAA is an apolipoprotein that forms HDL. In our study, glucose levels had the strongest correlation (inverse relationship) with the ratio of truncations in males, and this persisted after adjusting for changes in kidney function, age or BMI. This inverse relationship with hyperglycemia suggests that glucose might impair the peptidase activity. In one previous study, the clearance of truncated SAA at the *N*-terminus differed from native SAA suggesting that these truncations may impact SAA turnover [16].

Our findings also suggest that sex may independently modify the activity of this peptidase, leading to a lower truncated SAA to native SAA ratio. Of note, male sex was previously identified as a risk factor for both amyloidosis and diabetes complications. In a study of Familial Mediterranean Fever, the male to female ratio was significantly higher (2:1) in patients with reactive amyloidosis, [34]. In the Pittsburgh Epidemiology of Diabetes Complications Study, males had worse diabetic complications after long term follow up [35]. The role of these truncations in chronic diabetic complications merits further study.

We measured total SAA concentrations by a validated commercial ELISA [25–29]. Total SAA concentrations did not differ between the diabetic and non-diabetic groups and SAA concentrations did not correlate with the ratio of SAA1.1R ratio. Although a previous study [36] demonstrated that SAA concentrations were increased in type 2 diabetes with chronic kidney disease, we did not find increased total SAA concentrations in persons with diabetes.

The relative abundance of the SAA RS truncation is lower than that of SAA R, and there has been speculation that it might be a biomarker of certain diseases, such as renal cancer [12]. However, the strong correlation of R and RS truncations with the unmodified SAA 1.1 variant suggest that the same process affects the formation of both of these truncations in patients with and without diabetes (Table 6). This may argue against RS truncation as a selective marker of diabetes complications.

It is worth noting that MSIA cannot distinguish SAA form 1.5 from 1.1 because their molecular weight differs by only 1 Da, which makes these forms difficult to differentiate from each other on most mass spectrometers. The expression frequencies of SAA 1.4 and 1.5 are not known, but based on previous genetic studies, they are likely uncommon variants [37]. Given the lower relative abundance of SAA 2.1, 2.2 and 1.3 truncations, this study could be underpowered to detect differences in these variants between the diabetes and non-diabetes groups.

In conclusion, our findings indicate that truncated SAA is less common in diabetes. SAA truncations may modify SAA function and clearance. Measuring SAA truncations may help us better understand the susceptibility to reactive amyloidosis in chronic conditions.
